# The Nuclear Function of IL-33 in Desensitization to DNA Damaging Agent and Change of Glioma Nuclear Structure

**DOI:** 10.3389/fncel.2021.713336

**Published:** 2021-10-20

**Authors:** Yu-Han Chung, Qiu Qian, Hsin-Ying Huang, Wen-Tai Chiu, Chung-Shi Yang, Shun-Fen Tzeng

**Affiliations:** ^1^Department of Life Sciences, College of Bioscience and Biotechnology, National Cheng Kung University, Tainan, Taiwan; ^2^Institute of Biomedical Engineering and Nanomedicine, National Health Research Institutes, Miaoli, Taiwan; ^3^Department of Biomedical Engineering, National Cheng Kung University, Tainan, Taiwan

**Keywords:** glioma, interleukin-33, DNA repair, nuclear structure, temozolomide

## Abstract

Glioma, the most common subtype of primary brain tumor, is an aggressive and highly invasive neurologically tumor among human cancers. Interleukin-33 (IL-33) is considered as a dual functional cytokine, an alarmin upon tissue damage and a nuclear chromatin-associated protein. Despite that, IL-33 is known to foster the formation of the inflammatory tumor microenvironment and facilitate glioma progression, evidence showing nuclear IL-33 function is still poor. In this study using lentivirus-mediated IL-33 gene knockdown (IL33KD) and IL-33 overexpression (IL33oe) in rat C6 glioma cells and human glioma cell lines (U251MG and U87MG), we found that IL33oe-glioma cells had resistance to the insults of the alkylating agent, temozolomide (TMZ), possibly because of the increased expression of DNA repair genes (i.e., BRCA1, BRCA2, Rad51, FANCB, and FANCD) in IL33oe-glioma cells. Alternatively, examination of glioma nuclear shape from transmission electron microscopy (TEM) imaging analysis and immunofluorescence for histone protein H2A staining showed that IL33KD attenuated the abnormal cancerous nuclear characteristic, such as indentation, long clefts, and multiple nucleoids. Yet, IL33oe promoted the changes in glioma nuclear shapes, such as the formation of multiple lobes. We further found that histone proteins, H2A and H3, were reduced in IL33KD glioma cells. The non-histone DNA-binding nucleoproteins, the high mobility group A1 (HMGA1) and HMGA2, were also downregulated by IL33KD. In contrast, IL33oe increased H2A and H3 proteins and HMGA1 and HMGA2 in glioma cells. Altogether, the upregulation of nuclear IL-33 expression was along with an increase in the expression of DNA repair genes, contributing to the desensitization of glioma cells to DNA damaging agents. Moreover, nuclear IL-33 proteins in cooperation with chromatin-associated proteins regulate glioma nuclear structure, which might be crucial for glioma progression and malignancy.

## Introduction

Glioblastoma multiforme (GBM), also known as astrocytoma WHO grade IV, is the most common and aggressive form of central nervous system (CNS) brain tumor (Ostrom et al., 2017; Lapointe et al., 2018). The conventional treatment for glioma combines surgery with radiotherapy and daily intake by alkylating drug TMZ (Stupp et al., 2015). The glioma adjuvant therapy using the other alkylating agent, such as carmustine wafer and platinum-based drugs, is commonly used against GBM progression and recurrence (Lapointe et al., 2018). Yet, the survival rate for most GBM patients remains less than a year, although 5% of GBM patients survive about 5 years (Ostrom et al., 2017). Given the fact that the immunosuppressive glioma-associated microenvironment is an obstacle to reducing anti-glioma competency, recent advances made through preclinical studies and clinical trials on immunotherapeutic approach have received great attention on the promising treatment of glioma by immune checkpoint inhibition (Xu et al., 2020). Nevertheless, any anti-glioma therapy still confronts challenges in DNA repair-associated resistance.

Interleukin-33 (IL-33; IL-1F11), a multifaceted IL-1 superfamily member, is processed from the full-length proIL-33 by proteinases, i.e., calpain, esterases, and cathepsin G (Schmitz et al., 2005; Haraldsen et al., 2009). It has been known that the IL-33 gene, protein, and biological function are conserved in mammals (Martin, 2013; Arshad et al., 2016; Cayrol and Girard, 2018). The full-length IL-33 is considered to be present in the nucleus since it contains nuclear localization sequence (NLS) in its N-terminus (Schmitz et al., 2005; Martin, 2013). IL-33 in the outside of cells acts as a classic cytokine through the binding to its heterodimeric receptor complex consisting of IL-1 receptor-related protein ST2 and IL-1 receptor accessory protein (IL1RAcP) to involve many inflammation-associated diseases, such as asthma, rheumatological diseases, atherosclerosis, cancer, and cardiovascular diseases (Liu and Turnquist, 2013; Martin and Martin, 2016; Pinto et al., 2018). IL-33 is also involved in tumorigenesis and the development of vascular diseases (Liew et al., 2010). Through lentivirus-mediated IL33KD and its receptor ST2 downregulation, we were first to show that IL-33 plays a critical role in creating a favorable microenvironment for glioma cell growth by regulating the production of the growth factors and cytokines/chemokines (Fang et al., 2014). The relevant studies have demonstrated that IL-33 and IL1RAcP mRNA levels were associated positively with human glioma tumor grades and poor glioma prognosis in humans (Gramatzki et al., 2016; Zhang et al., 2017a). Later, it is increasingly evident to indicate IL-33/ST2 axis as the stimulator to promote glioma cell migration and invasion by the actions of MMP2, MMP9, and tenascin C (Zhang et al., 2017b, 2019). The IL-33/ST2-induced the c-Jun N-terminal kinase (JNK) activation has been also reported to mediate TMZ resistance (Lin et al., 2020). Recently, interesting findings have addressed that nuclear IL-33 was involved in the infiltration and recruitment of activated immune cells into tumor sites for the formation of the inflammatory glioma-associated environment (De Boeck et al., 2020).

Nuclear IL-33 is thought to be tightly associated with chromatin *via* its chromatin binding motif (45–53 residues) located at the amino-terminal nuclear domain to bind to the acidic pocket of the histone heterodimer H2A–H2B complex (Carriere et al., 2007; Roussel et al., 2008; Liew et al., 2016). To date, information about the nuclear function of IL-33 in glioma proliferation and tumor development is still limited. The chromatin interaction has been thought to provide a fine-tune regulation in IL-33 release and IL-33/ST2-mediated signaling upon cellular necrosis (Travers et al., 2018). Given that H2A and H2B are the critical structural proteins to mediate chromatin modeling and transcriptional activity, in this study, we proposed that nuclear IL-33 in glioma may contribute to chromatin modeling and mediate nuclear structure, triggering DNA replication and gene transcription. Through lentivirus-mediated IL-33 shRNA or IL-33 gene delivery to trigger IL-33 downregulation or increase IL-33 expression in glioma cells, we found that the alteration of nuclear IL-33 expression changed the sensitivity of glioma cells to TMZ. Moreover, nuclear IL-33 was involved in regulating the expression of histone proteins and chromatin-associated proteins, which might lead to the reshaping of nuclear/chromatin structure in glioma cells.

## Materials and Methods

### Cell Culture

The two rat glioma C6 cell lines (named as C6-1 and C6-2 in our laboratory) were originally obtained from the National Health Research Institute Cell Bank (Zhunan, Taiwan) or the American Type Culture Collection (ATCC; Manassas, VA, USA), respectively. The high tumorigenic C6-1 cells with enriched IL-33 expression and low tumorigenic C6-2 with extremely low IL-33 expression have been previously characterized *in vitro* and *in vivo* (Fang et al., 2014). These cells were grown in culture flasks in Dulbecco's Modified Eagle's medium (DMEM)/F12 (Gibco, Grand Island, NY, USA), containing 10% heat-inactivated fetal bovine serum (FBS; HyClone, Logan, UT, USA). Alternatively, human malignant glioma cell lines, U251MG and U87MG, originally from ATCC were maintained in DMEM medium (Gibco) containing 10% FBS. The culture medium and antibiotics were purchased from Invitrogen (Carlsbad, CA, USA).

### Lentivirus-Mediated Gene Delivery of shRNA Targeting IL-33

The shRNA-mediated rat and human IL33KD was performed using shRNA lentiviral particles (Biosettia Inc., San Diego, CA, USA). The cultures (1 × 10^6^ cells onto a 60-mm petri dish) that were transfected with shRNA lentiviral particles with non-effective scramble shRNA sequences were used as the control group (Scramble). The lentivirus vector constructs that have been used in our previous study (Fang et al., 2014) are listed as follows: pLV-mU6-[sh-scramble]EF1a-GFP-puromycin (Puro), pLV-mU6-[sh-rnoIL33]EF1a-Puro (lenti-shIL33_795), and pLV-mU6-[sh-hIL33] EF1a-Puro (lenti-sh-hIL33_661). The shRNA sequences are included in Table 1. The gene transduction into C6-1, U251MG, and U87MG cells was followed by the procedure as described previously (Fang et al., 2014) and selected in the presence of Puro (3 μg/ml) for 48 h. The cultures were maintained in the fresh medium containing 10% FBS. The efficiency of IL33KD in these glioma cell lines was validated by gel-based PCR or quantitative PCR (qPCR) analysis (Supplementary Figure 1).

**Table 1:** Primer sequences for qPCR analysis and sequences for shRNA against IL-33.

Rat IL-33 (NM_001014166)	Forward: 5^′^-GTGCAGGAAAGGAAGACTCG-3^′^ Reverse: 5^′^-TGGCCTCACCATAAGAAAGG-3^′^
Human IL-33 (NM_033439)	Forward: 5^′^-TGGCCTACCATCCCTTCTGACCC-3^′^ Reverse: 5^′^-CGTTTTGGCATCCAGAGGTCAGGT-3
Rat GAPDH (NM_017008)	Forward: 5^′^-TCTACCCACGGCAAGTTC-3 Reverse: 5^′^-GATGTTAGCGGGATCTCG-3
Human GAPDH (NM_002046)	Forward: 5^′^-TTGGTATCGTGGAAGGACTCA-3 Reverse: 5^′^-TGTCATCATATTTGGCAGGTTT-3
Rat BRCA1 (NM_012514.1)	Forward: 5^′^-ACCACACAACTTAACAGGGCG-3 Reverse: 5^′^-TCAGGGTCTCTGCTGAGAACTA-3
Rat Rad51 (NM_001109204.1)	Forward: 5^′^-AGTGTGTTTGAGCCGTGTGA-3 Reverse: 5^′^-GCCATTACTGTCTCGCAGCC-3
Human BRCA1 (NM_007294.4)	Forward: 5^′^-agcagaatggtcaactgatgaata-3 Reverse: 5^′^-ACTGCTGCTTATAGGTTCAGCTTT-3^′^
Human BRCA2 (NM_000059.4)	Forward: 5^′^- aattagcatgtgagaccattgaga-3^′^ Reverse: 5^′^-gatttgtgtaacaagttgcaggac-3
Human BRCC3 (NM_001025291)	Forward: 5^′^- CGTTTGTCTCAACCACGCTC-3 Reverse: 5^′^-TTGTATCATCGTTCAACTCCCC-3^′^
Human FANCB (NM_001018113.3)	Forward: 5^′^- TGTTGTTGGAGTGAAAACTACAT-3^′^ Reverse: 5^′^- ACAAAGCTTTCCTCTTTCTTGCTA-3^′^
Human FANCD (NM_033084.6)	Forward: 5^′^- AGAGTTGTGGATGGCAAGGAC-3^′^ Reverse: 5^′^- AAGTTTGGGTCAAGTCGGAGG-3^′^
Human Rad51 (NM_021770)	Forward: 5^′^-CAGTGATGTCCTGGATAATGTAGC-3 Reverse: 5^′^-ttacc actgctacaccaaactcat-3
Human HMGA1 (NM_145899.3)	Forward: 5^′^- GCTGGTAGGGAGTCAGAAGGAG-3^′^ Reverse: 5^′^- GTCTTGGCAGCACCCTTGTT-3^′^
Human HMGA2 (NM_003483.6)	Forward: 5^′^- TGCCACCCACTACTCTGTCC-3^′^ Reverse: 5^′^- CCCTTGAAATGTTAGGCGGG-3^′^
MOCK	pLVpuro-EF1a-F2A-GFP-Bsd
sh-rnoIL33_795	AAAAGGCTCTGGTAGAAGAGAATTT GGATCCAAATTCTCTTCTACCAGAGCC
sh-hIL33_661	AAAAGGAGTGTTTATAGGTGTAATTGG ATCCAATTACACCTATAAACACTCC
sh-scramble	AAAAGCTACACTATCGAGCAATTTTGGAT CCAAAATTGCTCGATAGTGTAGC
Full length IL-33 cDNA (NM_001014166)	LIC-BamHI-rnoIL33-Forward 5^′^-3^′^ atagggcccgggttggatccgccaccatgagacctagaatgaagtattcgaactcc LIC-NheI-rnoIL33-Reverse 5^′^-3^′^ ggggagggagaggggctagcttacatcttagagagcttaaacatgatattgttac (pLV-EF1a-rnoIL33IRES-puro)
IL33-FLAG	pLVPuro-EF1a-rnoIL33-3xFlag

*IL-33, Interleukin-33*.

### Generation of Rat and Human Glioma Cells With IL33oe

To generate glioma cells with IL33oe, 300 μl of recombinant lentivirus particles with pLV-EF1a-rnoIL33-IRES-Puro were also produced by Biosettia (San Diego, CA, USA) and transduced into 1 × 10^6^ C6-2 cells cultured onto a 60-mm petri dish with 3 ml 10% FBS-containing medium (Fang et al., 2014). Alternatively, C6-2, IL33oe-U251MG, and Il33oe-U87MG cells were generated by gene transduction of pLVpuro-EF1a-rnoIL33-3xFLAG (Biosettia) to express IL-33 with reporter peptide 3xFLGA. These IL33oe transfectants were selected in the presence of Puro (3 μg/ml) for 48 h and then maintained in the fresh medium containing 10% FBS. The cells transduced by pLVpuro-EF1a-GFP-Bsd were referred to be the control cells (MOCK). Total RNA was prepared and subjected to qPCR to determine the efficiency of IL33oe (Supplementary Figure 1).

### Quantitative Real-Time PCR

Total RNA was isolated with Trizol reagent (Invitrogen) from cells and converted to cDNA using Moloney Murine Leukemia Virus (M-MLV) reverse transcriptase (Invitrogen) with oligo (dT) as a primer. qPCR assays were performed on ABI StepOnePlus Real-Time PCR System (Applied Biosystems, Foster City, CA, USA) using FastStar Universal SYBR Green Master (Roche Diagnostics, Rotkreuz, Switzerland). PCR amplification of the genes analyzed in this study was performed for 10 min at 95°C, followed by 40 cycles at 95°C for 10 s, annealing at 60°C for 10 s, and extension at 72°C for 20 s as described previously (Fang et al., 2014; Sung et al., 2019). The level of the glyceraldehyde-3-phosphate dehydrogenase (GAPDH) mRNA expression in each culture was an internal control. As described in our previous study (Fang et al., 2014; Sung et al., 2019), the cycle threshold (Ct) fluorescence values were obtained *via* processing the data through StepOne Software v2.1 (Applied Biosystems). The expression level of the indicated genes relative to GAPDH level was presented as 2^−ΔCT^. ΔCT is equal to (Ct_target_ – Ct_GAPDH_). The results were presented as the percentage of IL33KD or IL33oe cultures over the relative control cultures, Scramble, or MOCK, respectively. The specific primer sequences for the genes analyzed in the study were designed using the Primer-BLAST, which was developed at NCBI. The primer sequences are shown in Table 1.

### Chromatin Fiber Preparation and Immunostaining

DNA spreads were prepared by adding cell suspension onto the microscopes followed by the protocol described previously (Nieminuszczy et al., 2016). The spread fibers were air-dried and then fixed by 4% paraformaldehyde for 15 min at room temperature. The samples were incubated with rabbit anti-IL-33 antibody and mouse anti-H1 (Table 2) overnight at 4°C and biotinylated conjugated anti-rabbit IgG and rhodamine-conjugated anti-mouse IgG (Table 2) for 1 h at room temperature. The cells were then incubated with avidin-Cy3 (Table 2) for 45 min at room temperature, and the samples were counterstained with 4′,6-diamidino-2-phenylindole (DAPI) and then mounted in 90% glycerol by coverslips. The amount of IL-33 punctate in the chromatic fiber with the length of 5 μm was counted.

**Table 2:** The antibodies used in the study.

**Antibody**	**Manufacturer**	**Dilution**
Mouse anti IL-33	Enzo RRID: AB_2124158	1:2000 (WB)
Rabbit anti IL-33	Santa Cruz Biotechnology RRID: AB_2124392	1:200 (IF)
Mouse anti GAPDH	Millipore (MAB374) RRID: AB_2107445	1:5000 (WB)
Mouse anti γH2AX	Abcam RRID: AB_26350	1:1000 (WB) 1:800 (IF)
Rabbit anti Lamin A/C	Cell Signaling Cat# 2032	1:2000~1:10000 (WB)
Mouse anti H1	Santa Cruz Biotechnology *RRID*:AB_675641	1:200 (IF)
Rabbit anti H2A	Abcam RRID: AB_20669	1:10000 (WB)
Mouse anti FLAG (DYKDDDDK)	Thermo Fishier Scientific *Cat# MA1*-*91878*.	1:1000 (WB)
Rabbit anti HMGA1	Cell signaling Cat# 7777	1:2500 (WB)
Rabbit anti HMGA1	Cell signaling Cat# 5269	1:2500 (WB)
Biotinylated anti-mouse IgG	Abcam RRID: AB_954888	1:200 (IF)
Biotinylated anti-rabbit IgG	Abcam RRID: AB_954902	1:200 (IF)
Biotinylated anti-goat IgG	Abcam RRID: AB_954842	1:200 (IF)
HRP-conjugated anti-mouse IgG	Jackson ImmunoResearch RRID: AB_10015289	1:2000 (WB)
HRP-conjugated anti-rabbit IgG	Jackson ImmunoResearch RRID: AB_2337938	1:2000 (WB)
Cy3- Streptavidin	Thermo Fisher Scientific Cat# 434315	1:800 (IF)
Alexa Fluor™ 488- Streptavidin	Thermo Fisher Scientific RRID: AB_2336881	1:200 (IF)
Rhodamine-conjugated anti-mouse IgG	Thermo Fisher Scientific RRID: AB_228308	1:200 (IF)
Alexa Fluor 488-conjugated anti-rabbit IgG	Thermo Fisher Scientific RRID: AB_2535792	1:200 (IF)

*IL-33, Interleukin-33*.

### Enzyme-Linked Immunosorbent Assay (ELISA) Assay for IL-33

The supernatants of MOCK- and IL33oe-C6 cell cultures were collected and centrifuged to remove cell debris. The samples (50 μl per well) were subjected for estimation of IL-33 levels in the culture medium using a mouse IL-33 ELISA assay kit (Abcam, Cambridge, UK). Absorbance was measured at 450 nm using an ELISA microplate reader (Thermo Fisher Scientific Multiskan, Singapore).

### Colony Formation Analysis

The cells were seeded onto 6-well plates (200 cells per well) and maintained in 10% FBS containing medium as previously described (Fu et al., 2018). At 5 days (Scramble- and IL33KD-C6) or 7 days (MOCK- and IL33oe-C6) after seeding, the cultures were treated by TMZ (100 and 200 μM) for 1 day. The cultures were then harvested by fixation in 4% paraformaldehyde and stained with 0.1% crystal violet solution. The colony number of each well was counted using NIH ImageJ software (https://imagej.nih.gov/ij/).

### 3-(4,5-Dimethylthiazol-2-yl)-2,5-Diphenyltetrazolium Bromide (MTT) Cell Viability Assay

U251MG cell growth was determined using a MTT colorimetric assay. Cells were seeded at a density of 5 × 10^4^ cells per well in 24 well plates in DMEM/F12 medium containing 10% FBS. After 24 h post-seeding, the cells were exposed to TMZ (200 μM) for 48 and 72 h, MTT solution (5 mg/ml, Sigma, Saint Louis, MO, USA) was added to each well for 4 h, followed by the addition of dimethyl sulfoxide to dissolve purple crystal precipitates. Absorbance was measured at 595 nm using an ELISA microplate reader.

### Western Blot Analysis

The total cell lysate was prepared by homogenizing the cells on ice in PBS containing 0.1% sodium dodecyl sulfate (SDS), 1 mM phenylmethylsulfonyl fluoride (PMSF), 1 mM ethylenediaminetetraacetic acid (EDTA), 1 mM sodium orthovanadate, and proteinase inhibitor cocktail (Sigma). Alternatively, nuclear protein extraction was conducted using a nuclear extraction kit (Cayman Chemical, Ann Arbor, MI, USA). The protein concentration of the supernatant was determined with a Bio-Rad DC protein assay kit (Bio-Rad Laboratories, Hercules, CA, USA). Nuclear proteins (20–40 μg/sample), cytoplasmic proteins (20–40 μg/sample), or total proteins (40–50 μg per sample) for the measurement of the desired proteins were separated on a 12.5 or 15% polyacrylamide gel and transferred to nitrocellulose membranes. The membranes were immunoblotted with a primary antibody and a secondary antibody conjugated with peroxidase (Table 2). The signals in the immunoblots were detected using the enhance chemiluminescence (ECL) kit (PerkinElmer Life Sciences, Waltham, MA, USA) by the iBright CL1500 imaging system (Thermo Fischer Scientific, Singapore) (for H2A, H3, and HMGA proteins in human glioma cell lines) or by exposure of immunoblots onto X-ray film (for γH2AX, IL-33, and GAPDH in C6 glioma cells). The information of primary antibodies used in this study is included in Table 2.

### Immunofluorescence

The procedure was followed by the method described previously (Sung et al., 2019). The cells were seeded at the density of 3 × 10^4^ cells/well onto the coverslips placed in a 24-well plate containing DMEM/F12 medium plus 10% FBS. After harvest, the cells were fixed with 4% paraformaldehyde for 15 min and treated 0.1% Triton X-100 in PBS for 10 min. The cells were incubated with primary antibody (Table 2) in PBS containing 1% horse serum (HS; Hyclone) at 4°C overnight. The cells were reacted with biotinylated secondary antibodies for 1 h at room temperature and avidin-Cy3 (or avidin-Alexa-488) for another 45 min. The nuclear counterstaining using DAPI (Thermo Fischer Scientific, Waltham, MA, USA) was also conducted. The immunoreactivity was observed under an Olympus FluoView laser-scanning confocal microscope (FV1000, Tokyo, Japan).

### TEM Analysis

MOCK- and IL33oe-U251MG cells were fixed in 2.5% glutaraldehyde in 0.1 M phosphate buffer (pH 7.4) for 1 h at 4°C, and post-fixed in 2% OsO_4_ in 0.1 M phosphate buffer for 1 h with light protection. The cells were dehydrated in 50, 70, 80, 90, and 100% ethanol, followed by embedded in Spurr's Resin. The sample blocks were then be sectioned, contrasted with heavy metals, and observed under Hitachi HT-7650 TEM imaging system (Tokyo, Japan) operated by the core facility of National Health Research Institutes (NHRI, Taiwan). Ten 1,500 × -magnification TEM images were digitally generated by an 11-Megapixel digital camera.

### Immunoprecipitation

Total proteins were isolated from 1 × 10^7^ MOCK- or IL33oe-C6 cells using cold PBS solution containing 2% Nonidet P-40, 1 mM PMSF, 1 mM EGTA, 1 mM sodium orthovanadate, and proteinase inhibitor (Sigma). The sample was mixed with an anti-FLAG antibody (Table 2) that was conjugated with dynabeads protein G (Thermo Fisher Scientific, Waltham, MA, USA) using a rotary shaker overnight at 4°C. After centrifugation, the immunoprecipitants were collected using magnet and heated in SDS-polyacrylamide gel electrophoresis (PAGE) gel loading buffer, and separated on a 12.5% polyacrylamide gel. After electrophoretic transfer to a nitrocellulose membrane, the membrane was incubated with an anti-FLAG antibody and horseradish peroxidase (HRP) anti-mouse IgG. After stripping, the membrane was incubated with rabbit anti-H2A antibody and HRP anti-rabbit IgG. The proteins were detected by incubating the immunoblots in the ECL reagent following by exposure to X-ray film for an optimal period.

### Statistical Analysis

All experiments were repeated at least three times, and data were analyzed for statistical significance by the two-tailed unpaired Student's *t*-test (Scramble vs IL33KD; MOCK vs IL33oe) and two-way ANOVA test with Tukey's comparisons for drug treatment and γH2AX quantification. The data are expressed as means ± SEM. In all comparisons, differences are considered statistically significant at *P* < 0.05.

## Results

### Nuclear IL-33 Expression and Interaction With DNA Fibers in C6 Glioma Cells

**Figure 1:**
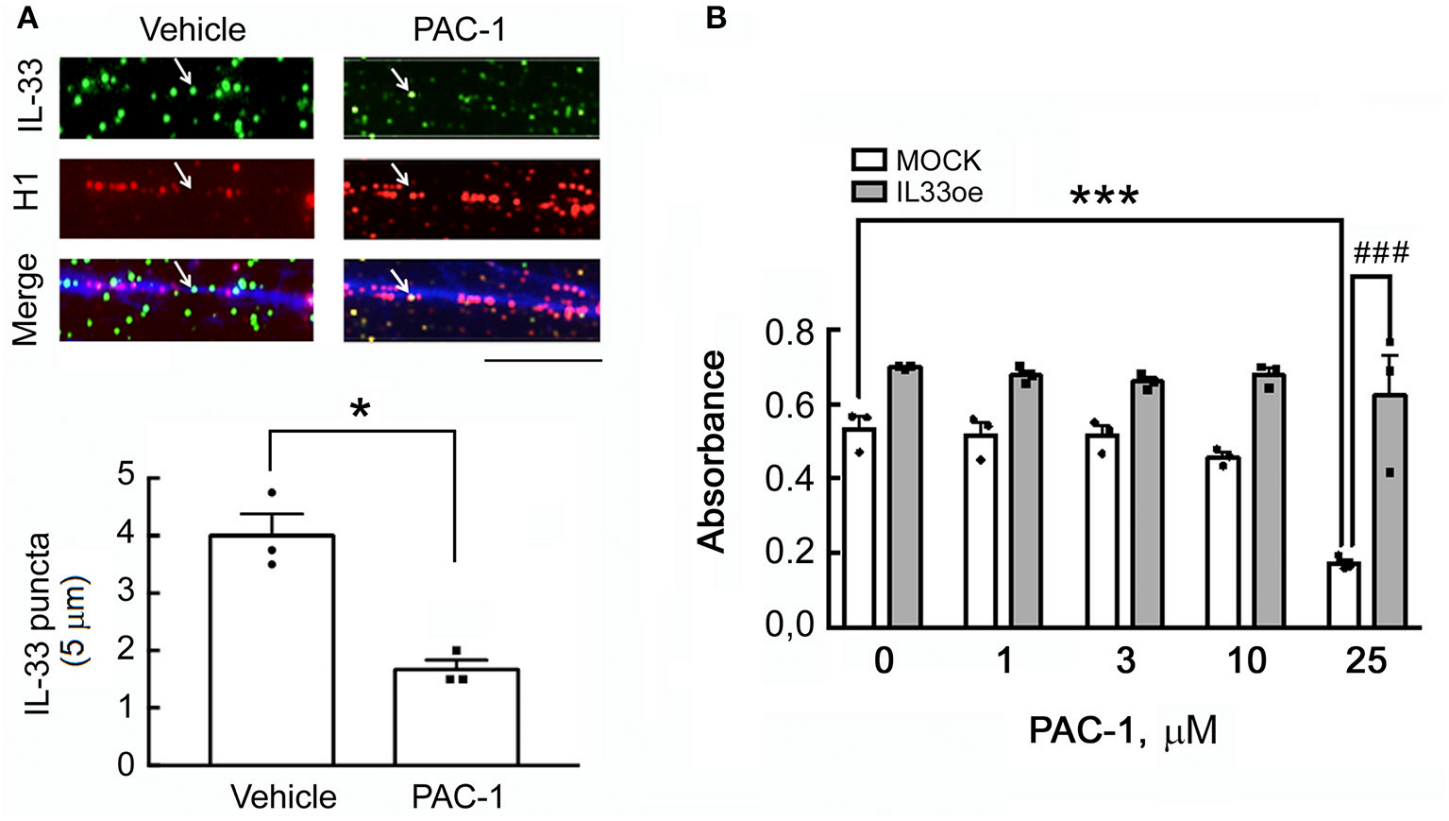
A caspase-3 activator, PAC-1, reduces IL-33 attachment to the chromatin fibers in IL33oe-C6 glioma cells but has no effect on the cell viability of IL33oe-C6 cells. **(A)** The chromatin fibers were prepared from IL33oe-C6 glioma cells at 24 h after exposure to 25 μM of PAC-1 for 24 h, and then subjected to double immunofluorescence for IL-33 (green; arrow), histone H1 (red), and DAPI counterstaining (blue). IL-33 puncta in chromatin fibers (5-μm length each) were counted manually and graphed. The results are presented as Mean ± SEM from the three cell passages (*n* = 3). *P < 0.05 vs. Vehicle. Scale bar, 5 μm. **(B)** MOCK- and IL33oe-C6 cells were treated with PAC-1 at the indicated concentrations for 24 h, and then examined their cell viability using MTT assay. The results are presented as Mean ± SEM from the three cell passages (*n* = 3). ***P < 0.001 vs. MOCK without PAC-1 treatment; ^###^*P* < 0.001 vs. MOCK in the presence of 25 μM of PAC-1. IL-33, Interleukin-33; IL33oe, IL-33 overexpression; DAPI, 4′ ,6-diamidino-2-phenylindole; MTT, 3-(4,5-Dimethylthiazol-2-yl)-2,5-diphenyltetrazolium bromide.

We first generated IL33oe-C6 cells by the lentiviral delivery of rat IL-33 cDNA into the C6-2 cell line that originally had extremely low expression of IL-33 (Fang et al., 2014). As shown in Supplementary Figure 2A, nuclear IL-33 was clearly observed in the nuclei of IL33oe-C6. In addition, we found that IL-33 molecules were only detected in the medium of IL33oe-C6 cells when the cells were lysed by 0.01% Triton X-100 (Supplementary Figure 2C), but rarely detected in the medium of IL33oe-C6 cells without exposure to 0.01% Triton X-100. The observations indicated that IL-33 released into the outside of the cells was not able to occur in the intact glioma cells unless the cells were destructed (Supplementary Figure 2C). Given that IL-33 protein can be processed by the action of caspase-3 (Luthi et al., 2009; Liew et al., 2016), the caspase-3 activator, PAC-1, was used to investigate the interaction of IL-33 proteins with the chromatin fibers in IL33oe-C6 cells. The results showed that IL-33 molecules interacted with the chromatin fibers indicated by histone H1 immunofluorescent staining combined with DAPI staining (Figure 1A, arrows). The amount of IL-33 interacting with the chromatin fibers was decreased upon exposure to PAC-1 for 24 h. Interestingly, IL33oe-C6 cells exhibited resistance to PAC-1-induced cell death, whereas exposure to 25 μM of PAC-1 significantly reduced the cell viability of the MOCK-C6 cells (Figure 1B; Table 3). We also noted that IL-33 was hardly detected in the culture medium (data not shown).

**Table 3:** *F* (with degrees of freedom) and *P*-values in two-way ANOVA test for experiments shown in Figures 1B, 2A,B, 3.

**ANOVA**	***F* (DFn, DFd)**	***P*-value**
Figure 1B		
Interaction	*F*_(4, 20)_ = 5.472	*P* = 0.0038
MOCK vs. IL33oe	*F*_(1, 20)_ = 86.39	*P* < 0.0001
Vehicle vs. PAC-1	*F*_(4, 20)_ = 10.42	*P* < 0.0001
Figure 2A		
Interaction	*F*_(2, 12)_ = 5.211	*P* = 0.0235
Scramble vs. IL33KD	*F*_(1, 12)_ = 45.28	*P* < 0.0001
Vehicle vs. TMZ	*F*_(2, 12)_ = 8.631	*P* = 0.0048
Figure 2B		
**γH2AX, 48 h**		
Interaction	*F*_(1, 8)_ = 13.94	*P* = 0.0058
MOCK vs. IL33oe	*F*_(1, 8)_ = 8.065	*P* = 0.0218
Vehicle vs. TMZ	*F*_(1, 8)_ = 21.26	*P* = 0.0017
**γH2AX, 72 h**		
Interaction	*F*_(1, 8)_ = 48.21	*P* = 0.0001
MOCK vs. IL33oe	*F*_(1, 8)_ = 47.28	*P* = 0.0001
Vehicle vs. TMZ	*F*_(1, 8)_ = 625.8	*P* < 0.0001
Figure 3		
**IL33KD-48 h**		
Interaction	*F*_(1, 8)_ = 0.01036	*P* = 0.9214
Scramble vs. IL33KD	*F*_(1, 8)_ = 68.36	*P* < 0.0001
Vehicle vs. TMZ	*F*_(1, 8)_ = 21.38	*P* = 0.0017
**IL33KD-72 h**		
Interaction	*F*_(1, 8)_ = 25.65	*P* = 0.0010
Scramble vs. IL33KD	*F*_(1, 8)_ = 690	*P* < 0.0001
Vehicle vs. TMZ	*F*_(1, 8)_ = 77.44	*P* < 0.0001
**IL33oe-48 h**		
Interaction	*F*_(1, 8)_ = 0.0004621	*P* = 0.9834
MOCK vs. IL33oe	*F*_(1, 8)_ = 54.84	*P* < 0.0001
Vehicle vs. TMZ	*F*_(1, 8)_ = 43.73	*P* = 0.0002
**IL330e-72 h**		
Interaction	*F*_(1, 8)_ = 17.38	*P* = 0.0031
MOCK vs. IL33oe	*F*_(1, 8)_ = 107.5	*P* < 0.0001
Vehicle vs. TMZ	*F*_(1, 8)_ = 33.58	*P* = 0.0004

*IL-33, Interleukin-33; IL33oe, IL-33 overexpression; TMZ, temozolomide*.

### Increased Resistance to TMZ in Glioma Cells With IL33oe

**Figure 2:**
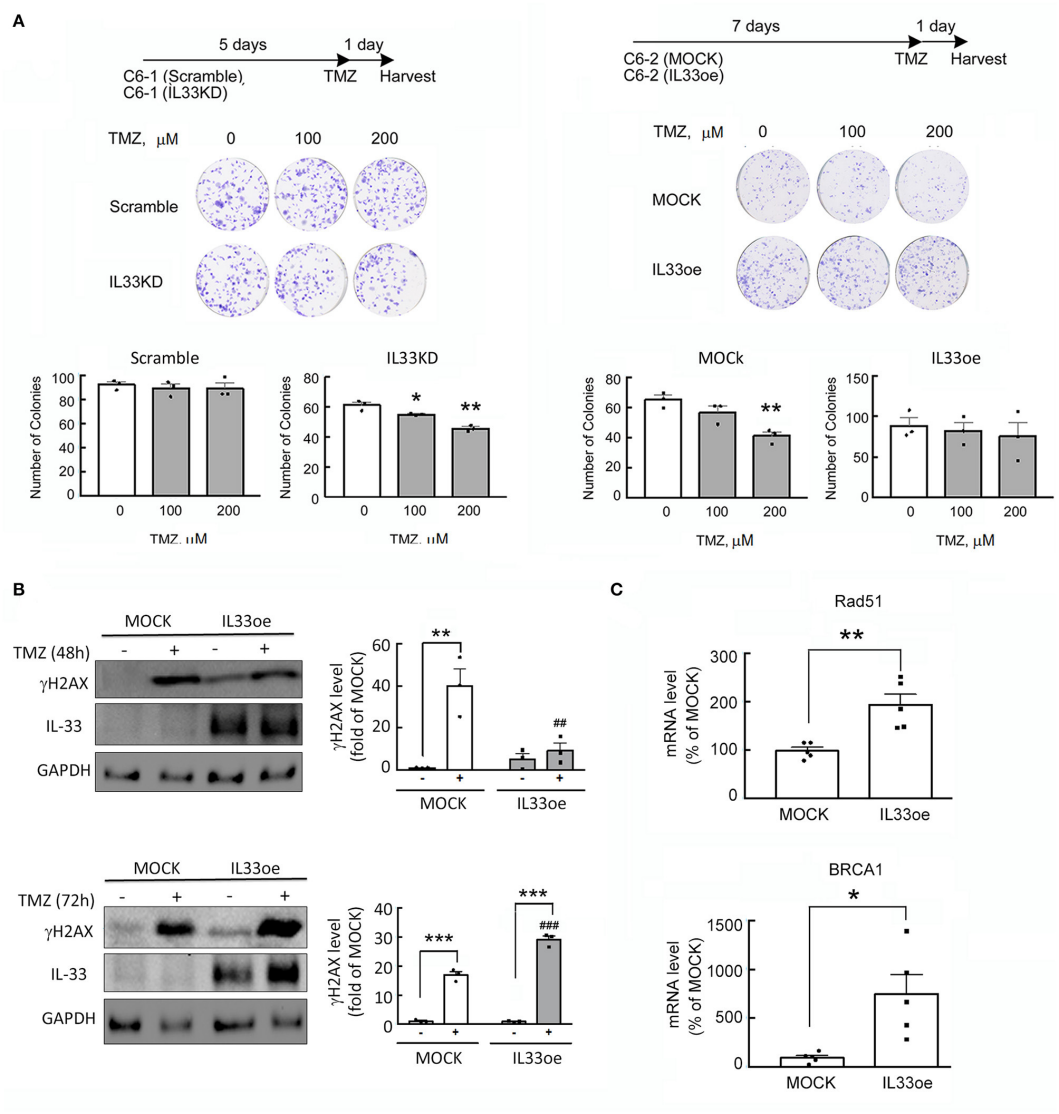
IL-33 protects glioma cells from the insult of DNA damaging drug, TMZ. **(A)** Scramble- and IL33KD-C6 cells were seeded onto a 6-well plate at the amount of 200 cells per well, and then grown in 10% serum containing medium for 5 days. The cultures were exposed to TMZ (0, 100, and 200 μM) for another day. Alternatively, MOCK- and IL33oe-C6 cells were maintained in 10% serum containing medium for 7 days, and treated with TMZ (0, 100, and 200 μM) for another day. The colonies were quantified using NIH Imaging J. Data are shown as mean ± SEM from the three cell passages (*n* = 3). *P < 0.05, **P < 0.01 vs. Vehicle. **(B)** MOCK- and IL33oe-C6 cells were treated without or with TMZ at 200 μM for 48 and 72 h. Total proteins collected from the cultures were subjected to Western Blot assay to examine the levels of γH2AX, IL-33, and GAPDH. The γH2AX protein levels in each immunoblot were normalized by the level of GAPDH (a loading control). The results are presented as mean ± SEM from three cell passages (*n* = 3). **P < 0.01, ***P < 0.001 vs. the relative control culture; ^##^*P* < 0.01, ^###^*P* < 0.001 vs. MOCK. **(C)** The expression of Rad51 and BRCA1 mRNA in MOCK- and IL33oe-C6 were measured by qPCR analysis. The results are presented as mean ± SEM from the five cell passages (*n* = 5). *P < 0.05, **P < 0.01 vs. MOCK. TMZ, temozolomide; qPCR, quantitative PCR.

The addition of recombinant IL-33 proteins has been found to induce glioma cells in resistance to TMZ-triggered apoptosis through ST2-mediated signal pathways (Lin et al., 2020). Here, we also observed that the colony formation ability of high tumorigenic C6 cells with abundant expression of IL-33 (Scramble-C6) was not affected by the treatment with TMZ (Figure 2A), while exposure of IL33KD-C6 cells to TMZ significantly reduced the number of glioma cell colonies when compared to that observed in Scramble-C6 either without or with TMZ treatment (Figure 2A; Table 3). Alternatively, the addition of TMZ had no effect on the colony number of IL33oe-C6 cells, although TMZ exposure caused the declined colony formation of MOCK-C6 cells to some extend (Figure 2A). We noticed that the number of IL33oe-C6 cell colonies was higher than that of MOCK-C6 cell colonies, although it was not bio-statistically different. However, the findings that the size of IL33oe-C6 cell colonies was larger than that observed in MOCK-C6 cultures were consistent with our previous results (Fang et al., 2014). In parallel, we found that the levels of γH2A expression in IL33oe-C6 cells were significantly increased at 72 h post-exposure to TMZ, while an increase in γH2A levels was observed in MOCK-C6 cells was earlier at 48 h after exposure to TMZ (Figure 2B; Supplementary Figure 2D; Table 3). In our previous study, we have shown that high tumorigenic C6 cells expressed high levels of DNA repair genes (Chai et al., 2014). Here, examination of DNA repair genes, Rad51 and BRCA1, indicated that the two-gene expression was upregulated in IL33oe-C6 cells compared to those detected in MOCK-C6 cells (Figure 2C). These results reveal that IL33oe caused an upregulation of DNA repair genes in C6 cells, which might contribute to TMZ resistance in C6 cells.

**Figure 3:**
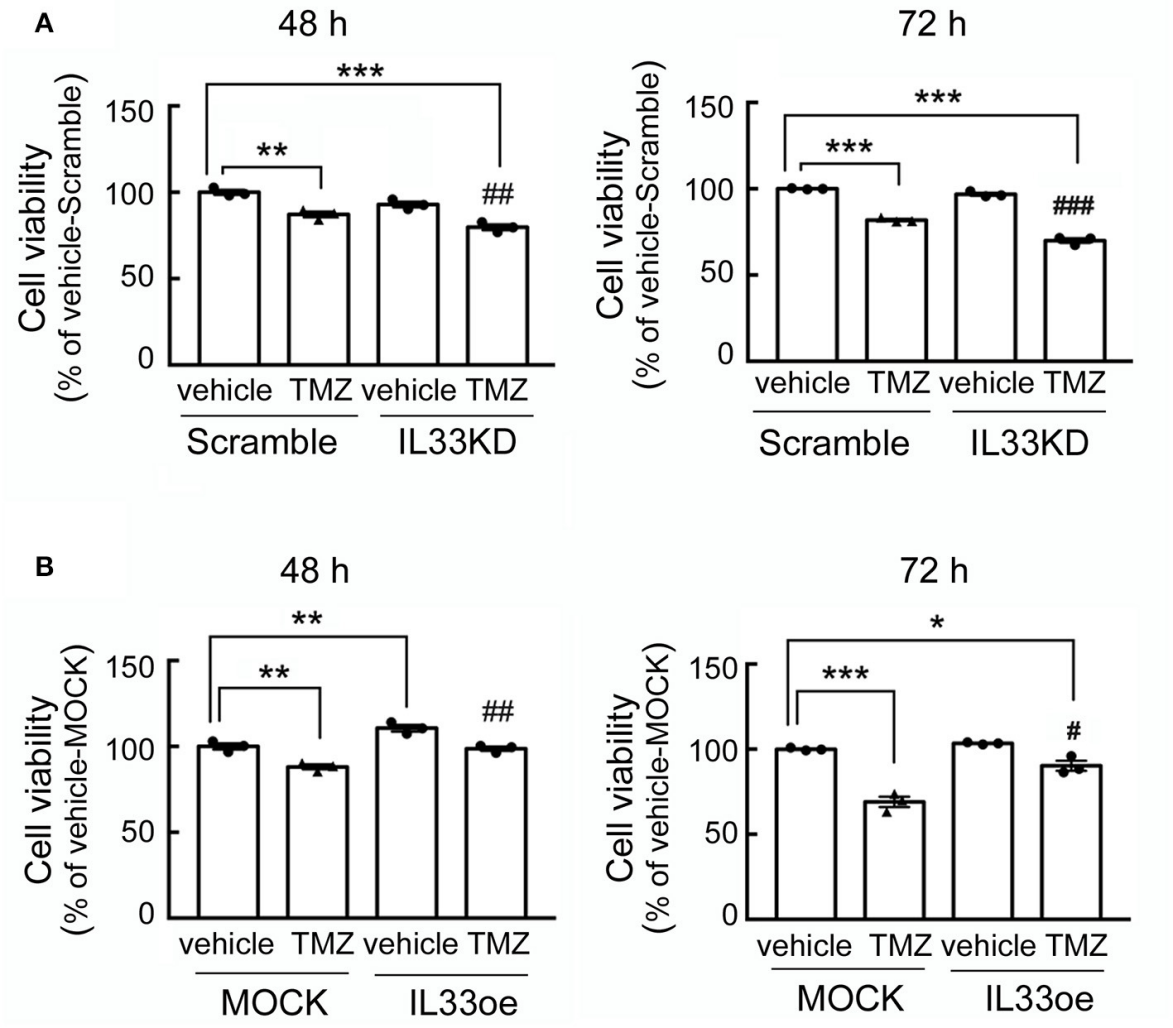
IL33oe increases the resistance of U251MG cells to the DNA damaging agent, TMZ. Scramble- and IL33KD-U251MG cells were treated by 200 μM of TMZ for 48 and 72 h **(A)**. Moreover, exposure of MOCK- and IL33oe-U251MG cells to 200 μM of TMZ was performed for 48 and 72 h **(B)**. The cell cultures at the end of the experimental time points were then subjected to MTT cell viability assay. Note that the cell density of control cultures at 72 h (Vehicle-Scramble, Vehicle-IL33KD, Vehicle-MOCK, and Vehicle-IL33oe) reached the levels of the confluency. Thus, the cell viability of these controls was insignificantly different. The results are presented as mean ± SEM from the three cell passages (*n* = 3). *P < 0.05, **P < 0.01, ***P < 0.001 vs. Vehicle-Scramble in A or Vehicle-MOCK in **(B)**. ^#^*P* < 0.05, ^##^*P* < 0.01, ^###^*P* < 0.001 vs. TMZ-Scramble in **(A)** or TMZ-MOCK in **(B)**. IL33oe, IL-33 overexpression; MTT, 3-(4,5-Dimethylthiazol-2-yl)-2,5-diphenyltetrazolium bromide; TMZ, temozolomide.

**Figure 4:**
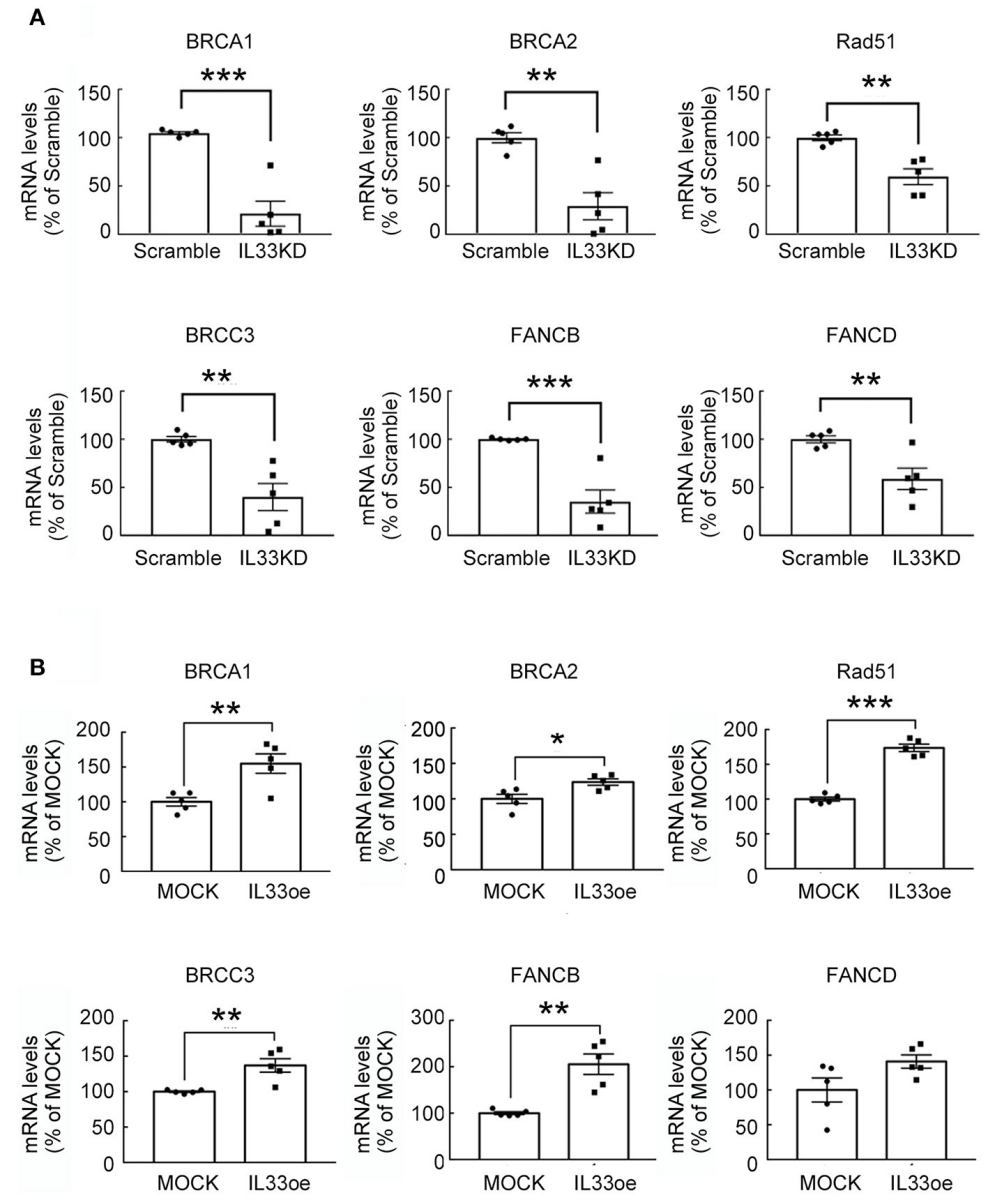
IL-33 regulates DNA repair genes in U251MG cells. RNA samples isolated from Scramble- and IL33KD-U251MG cells **(A)**, as well as MOCK- and IL33oe-U251MG cells **(B)**, were subjected to qPCR analysis for the measurement of DNA repair genes (i.e., BRCA1, BRCA2, Rad51, BRCC3, FANCB, and FANCD). The results are present as mean ± SEM from the five cell passages (*n* = 5). *P < 0.05, **P < 0.01, ***P < 0.001 vs. Scramble **(A)** or MOCK **(B)**. IL-33, interleukin-33.

**Figure 5:**
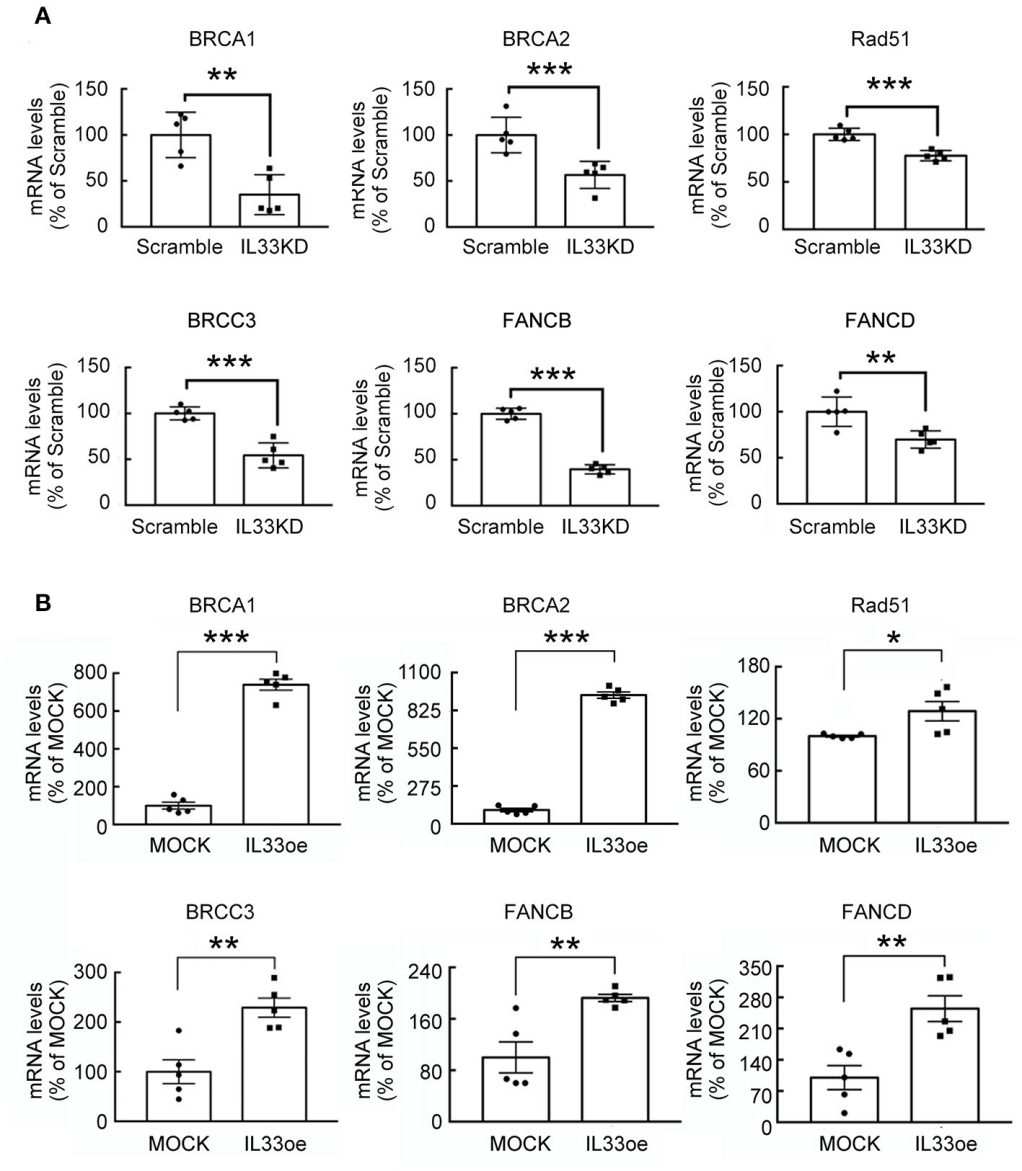
DNA repair gene expression is mediated by IL-33 in U87MG cells. RNA samples were prepared from Scramble and IL33KD-U87MG cells **(A)**, as well as MOCK- and IL33oe-U87MG cells **(B)**. The expression of DNA repair genes (BRCA1, BRCA2, Rad51, BRCC3, FANCB, and FANCD) was measured by qPCR analysis. The results are present as mean ± SEM from the five cell passages (*n* = 5). *P < 0.05, **P < 0.01, ***P < 0.001 vs. Scramble **(A)** or MOCK **(B)**. IL-33, interleukin-33; qPCR, quantitative PCR.

The observations as shown above propelled us to investigate if IL33oe induced DNA repair mechanism to protect human glioma cells from the destruction of DNA damaging drugs. Thus, we further investigated TMZ-induced reduction in glioma cell viability and how it may have changed after the altered expression of nuclear IL-33 in human 251MG cells. We found that IL33KD promoted TMZ-induced inhibition in U251MG cell proliferation (Figure 3A; Table 3). Yet, IL33oe in U251MG induced resistance to the inhibitory effect of TMZ on their cell proliferation (Figure 3B; Table 3). Further experiments showed that the expression of DNA repair genes, such as BRCA1, BRCA2, Rad51, BRCC3, FANCB, and FANCD, declined significantly in IL33KD-U251MG cells (Figure 4A), whereas these gene expressions were increased in IL33oe-U251MG cells (Figure 4B). Similarly, IL33KD reduced the expression of the indicated genes in U87MG cells (Figure 5A), and IL33oe upregulated these gene expressions in U87MG cells (Figure 5B). Thus, the positive regulatory function of IL-33 in the glioma DNA repair system might lead to the desensitization of glioma cells with high IL-33 expression to the insult of DNA damaging agents.

### Expression of Histone Proteins and HMGA Proteins in Glioma Cells Mediated by IL-33

**Figure 6:**
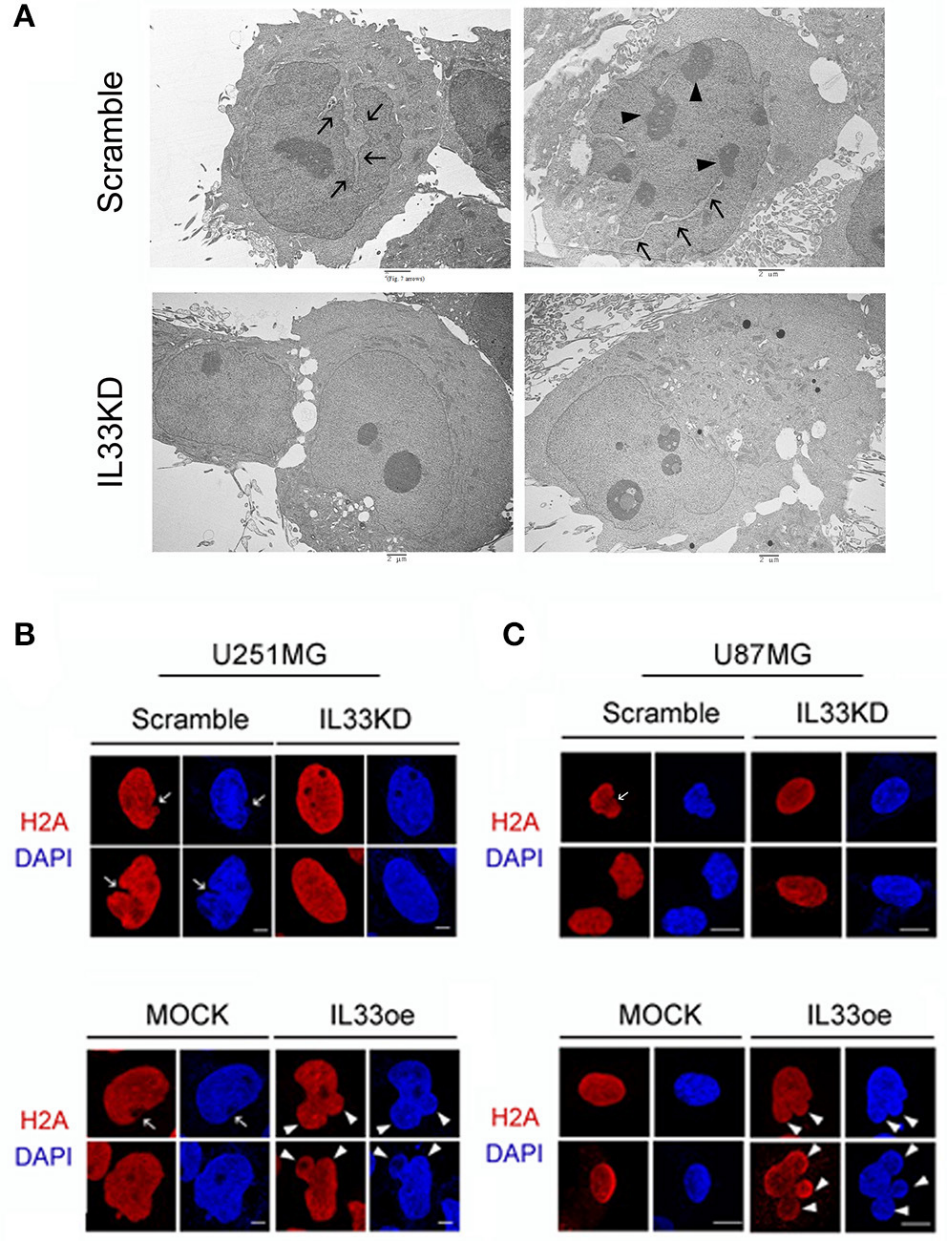
Altered expression of nuclear IL-33 causes a change in the nuclear shape of human glioma cells. **(A)** Scramble- and IL33KD-U251MG cells were prepared for TEM imaging examination. Representative TEM images show a nucleus with grooves and long clefts (arrows), or with multiple nucleoli (arrowheads). Similar results were observed in the different passage cell cultures. Scale bar, 2 μm. **(B)** Representative confocal images of immunofluorescent staining for H2A (red) and DAPI counterstaining (blue) show cancerous nuclear groove and cleft in Scramble- and MOCK-U251MG cultures (arrow), and indicate multiple nuclear lobes in IL33oe-U251MG cultures (arrowheads). **(C)** Representative confocal images of H2A immunofluorescence H2A (red) and DAPI counterstaining (blue) indicate nuclear cleft in Scramble-U87MG cells (arrow) when compared to those seen in IL33KD-U87MG cells. In addition, polylobulation in IL33oe-U87MG cultures is indicated by arrowheads. The experiments were repeated with similar results. Scale bar in **(B,C)**, 10 μm. IL-33, interleukin-33; DAPI, 4',6-diamidino-2-phenylindole.

Examination of U251MG cell nuclear shape by TEM imaging indicated that the nucleus structure with grooves and long clefts (Figure 6A, arrows), and multiple nucleoli (Figure 6A, arrowheads), were observed in Scramble-U251MG cells, but less seen in IL33KD-U251MG cells. Nuclear IL-33 is a chromatin-associated protein by its binding to H2A and H2B acidic patch (Roussel et al., 2008). In conjunction with our observations through a pull-down assay using an anti-FLAG showing that H2A proteins were able to be detected in anti-FLAG immunoprecipitants of IL33oe-C6 cells (Supplementary Figure 2B), we further conducted experiments by immunofluorescence for H2A in U251MG and U87MG cells. The results showed that IL33KD reduced cancerous nuclear groove and cleft (Figure 6B, arrows), whereas IL33oe promoted the nuclear abnormality with the lobe structure (Figure 6B, arrowheads). Similarly, IL33oe-induced multiple lobe formation (polylobulation) was also observed in U87MG cell nuclei (Figure 6C, arrowheads). The findings suggest that nuclear IL-33 proteins might mediate the nuclear shape through their interaction with histone proteins and chromatins.

**Figure 7:**
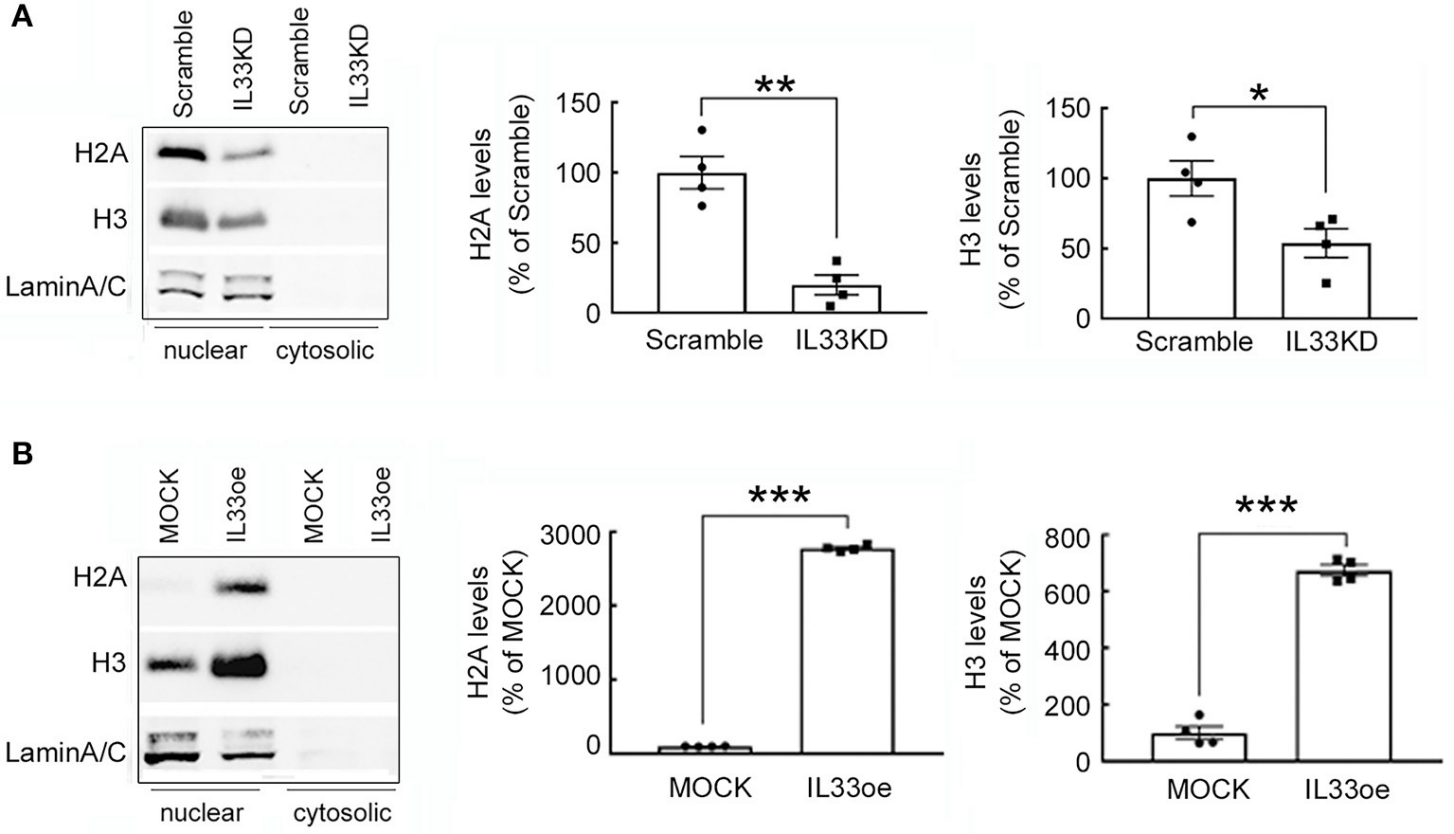
H2A and H3 protein expression is regulated by IL-33 in human glioma cells. Nuclear proteins and cytosolic proteins were isolated from Scramble- and IL33KD-U251MG cells **(A)**. Additionally, the protein samples were prepared from MOCK- and IL33oe-U251MG cells **(B)**. Western Blot analysis was conducted to measure the nuclear levels of H2A, H3, and Lamin A/C (as a loading control) in these transfectants. Representative images show the three proteins were only detected in the nuclear fraction. The nuclear levels of H2A and H3 in each immunoblot were quantified and normalized by the level of Lamin A/C. The results are presented as mean ± SEM from the four cell passages (*n* = 4). *P < 0.05, **P < 0.01, ***P < 0.001 vs. Scramble in **(A)** or MOCK in **(B)**. IL-33, interleukin-33.

Furthermore, examination of histone proteins (H2A and H3) in U251MG cells revealed that the expression levels of H2A and H3 proteins were significantly lower in the nuclear fractions of IL33KD-U251MG cells compared to that in the Scramble group (Figure 7A). In contrast, nuclear H2A and H3 proteins were much higher in IL33oe-U251MG cells than that observed in MOCK-U251MG cells (Figure 7B). Note that H2A and H3 proteins were mainly detected in the nuclear fractions of Scramble-/IL33KD- or MOCK-/IL33oe-glioma cells (Figure 7). Similarly, H2A and H3 proteins in IL33KD-U87MG cells were toward a decreased level. Moreover, their levels in IL33oe-U87MG cells were increased when compared to those observed in MOCK-U87MG cells (Supplementary Figure 3A).

**Figure 8:**
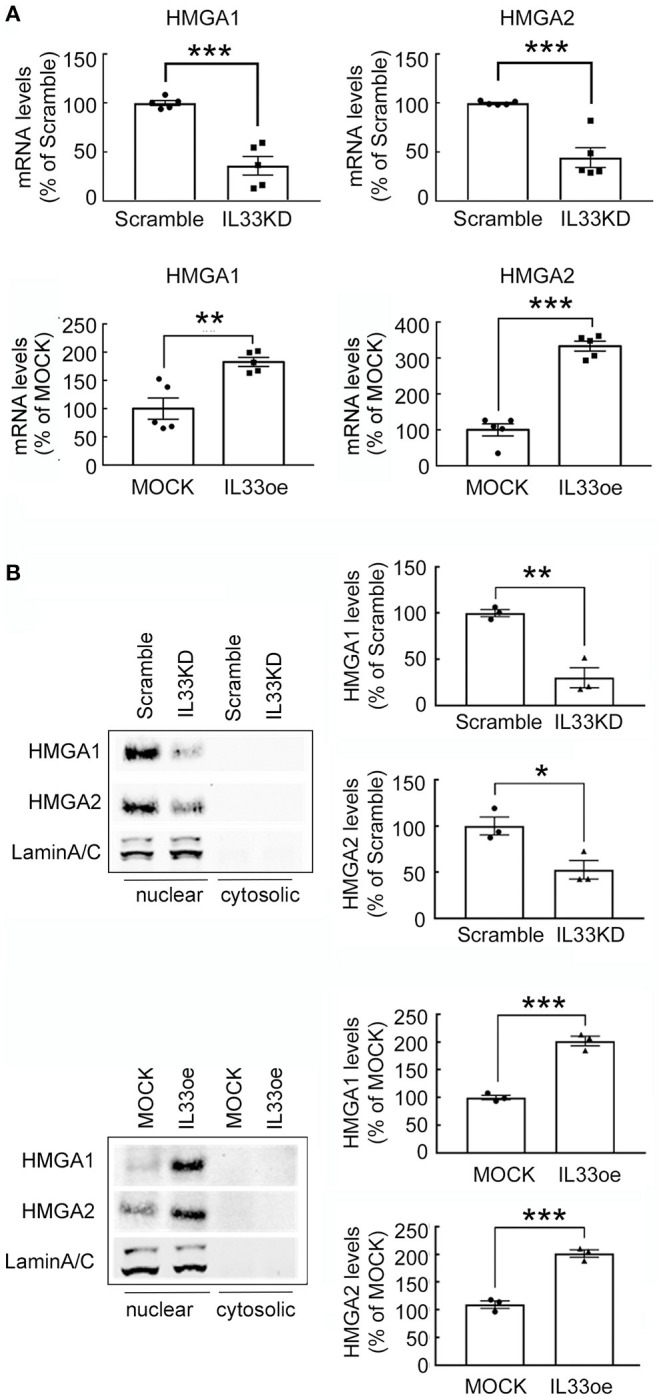
Expression of non-histone proteins HMGA1 and HMGA2 is mediated by IL-33 in U251MG cells. **(A)** HMGA1 and HMGA2 mRNA expression in Scramble-, IL33KD-, MOCK, and IL33oe-U251MG cells was examined by qPCR analysis. The results are presented as mean ± SEM from five cell passages (*n* = 5). **P < 0.01, ***P < 0.001 vs. Scramble or MOCK. **(B)** Nuclear proteins and cytosolic proteins were isolated from Scramble- and IL33KD-U251MG cells, as well as MOCK- and IL33oe-U251MG cells. Representative images of the Western Blot analysis show the nuclear levels of HMGA1, HMGA2, and Lamin A/C in the transfectants. The nuclear levels of HMGA1 and HMGA2 proteins were normalized by the level of Lamin A/C (as a loading control). The results are presented as mean ± SEM from the three cell passages (*n* = 3). *P < 0.05, **P < 0.01, ***P < 0.001 vs. Scramble or MOCK. IL-33, interleukin-33; IL33oe, IL-33 overexpression; HMGA1, the high mobility group A1; HMGA2, the high mobility group A2; qPCR, quantitative PCR.

High mobility group A proteins, so-called architectural transcription factors binding to the minor groove of AT-rich DNA, can remodel chromatin structures and contribute to cancer transformation (Fusco and Fedele, 2007). Accordingly, HMGA1 and HMGA2 expression was investigated in human glioma cells after IL33KD or IL33oe. We found that HMGA1 and HMGA2 mRNA expression was downregulated by IL33KD, but upregulated by IL33oe in U251MG cells (Figure 8A). Similar to the alteration of nuclear H2A and H3 levels in U251MG cells, the expression of HMGA1 and HMGA2 proteins mainly found in the nuclear fractions of U251-MG cells was decreased by IL33KD but increased by IL33oe (Figure 8B). The change in HMGA2 mRNA expression and HMGA2 protein production was observed in IL33KD- and IL33oe-U87MG cells (Supplementary Figures 3B, 4), although HMGA1 mRNA expression was not affected in U87MG cells by IL33KD or IL33oe. Overall, these results reveal that nuclear IL-33 could mediate the chromatin remodeling by the regulation of chromatin structural proteins.

## Discussion

The study demonstrates that the upregulation of nuclear IL-33 levels in glioma cells promoted the expression of DNA repair genes, which could play a critical role in the desensitization of IL33oe-glioma cells to the DNA damaging agent TMZ. In addition, the downregulation of nuclear IL-33 was in concert with declined levels of histone proteins and non-histone chromatin-associated proteins in glioma cells. The overexpression of nuclear IL-33 was along with increased expression of histone proteins and non-histone chromatin-associated proteins in glioma cells. The findings demonstrate that nuclear IL-33 participates in the regulation of glioma cell nuclear architecture.

Interleukin-33 acts as a secreted cytokine and binds to the ST2 receptor which can trigger MyD88/IRAK/TRAF6 signal cascades, NFκB signaling, JAK2/STAT3, and PI3K-AKt-mTOR pathway (Martin, 2013; Pinto et al., 2018). Yet, the evidence provides here that IL-33 was rarely detected in the cultured medium of the intact IL33oe-C6 cells unless IL33oe-C6 cells were damaged by 0.01% Triton X-100. Moreover, exposure to the caspase-3 activator PAC-1 did not affect the cell viability of IL33oe-C6 cells, and IL-33 molecules were undetectable in the cell culture of IL33oe-C6 cells after treatment with PAC-1 (data not shown). These observations are consistent with the findings that IL-33 was not released from cells undergoing the apoptotic process, unless the cellular destruction or necrosis occurred (Ali et al., 2010). Moreover, the chromatin-associated IL-33 has been proposed to have low bioavailability for release (Travers et al., 2018). Accordingly, exposure of glioma cells to PAC-1 did decrease the amount of IL-33 molecules attached to the DNA fibers, implicating that IL-33 molecules detached from the chromatins might remain in the intracellular matrix.

The deficiency of IL-33/ST2 signaling has been suggested to be involved in the accumulation of DNA double-strand breaks observed in the cortical and hippocampal neurons in aged IL-33^−/−^ mice (Carlock et al., 2017). The findings have also pointed to the critical role of IL-33 in DNA repair initiation of aged neurons (Carlock et al., 2017). Through the examination of glioma microenvironment-associated secretome in the interstitial fluid from glioma patient-derived brain tumor-initiating cell (BTIC) orthotropic animals, an abundant amount of IL-33 was found in the secretome of xenografts receiving highly invasive rapidly growing tumors (De Boeck et al., 2020). We have previously reported that the downregulation of ST2 suppressed the expansion of glioma cells (Fang et al., 2014). Exogenous IL-33/ST2 axis has been shown to contribute to the prevention of TMZ-induced glioma cell apoptosis through the JNK signaling pathway (Lin et al., 2020). Nevertheless, the differential effect of PAC-1 on the cell viability of MOCK- and IL33oe-C6 cells raised the possibility that IL33oe might protect glioma cells from the insult of DNA damaging drugs. Indeed, we observed that IL33KD promoted TMZ-induced cell damage in rat C6 glioma cells and human U251MG cells, whereas IL33oe can prevent glioma cells from the death induced by TMZ. Thus, the nuclear IL-33 might be involved in the protective pathway in the desensitization of glioma cells to DNA damaging agents. This suggestion is also supported by the findings that an increase in γH2AX was detected in MOCK-C6 cells treated by TMZ for 48 h and 72 h, whereas the delayed elevated expression of γH2AX was observed in IL33oe-C6 cells for a 72-h exposure to TMZ. Besides, DNA repair gene expression was significantly upregulated in IL33oe-glioma cells when compared to those examined in MOCK-glioma cells. Nevertheless, our findings in conjunction with others as mentioned above indicate that the expansion of glioma cells is not only stimulated by exogenous IL-33 released by injured brain cells or from circulating immune cells through the ST2-triggering signaling pathway, but also enhanced by the action of their nuclear IL-33.

Nuclear IL-33 has been reported to have no impact on global gene expression in the three clones of the epithelial cells with IL33oe (Travers et al., 2018). Yet, nuclear IL-33 has been considered as a transcriptional factor to repress or induce gene expression (Carriere et al., 2007; Kuchler et al., 2008; Haraldsen et al., 2009). Our previous study has addressed that IL33KD caused the differential expression of the distinct gene clusters, such as reduction in inflammatory genes in glioma cells (Fang et al., 2014) and glia differentiation-associated genes in oligodendrocyte precursor cells (Sung et al., 2019). Here, we show that IL33KD reduced DNA repair gene expression in two human glioma cell lines. However, IL33oe upregulated the expression of DNA repair genes in rat glioma cell line and two human glioma cell lines. Given that nuclear IL-33 is considered as a chromatin-associated nuclear factor (Carriere et al., 2007), our results regarding IL-33-mediated DNA repair gene expression, in conjunction with others, propose an explanation that the altered expression of nuclear IL-33 may result in the modification of chromatin structure and subsequently enable to mediate transcription (Haraldsen et al., 2009). Yet, the upregulation of nuclear IL-33 expression in glioma cells induced the gene expression and protein secretion of inflammatory cytokines and chemokines such as IL-1β, IL-6, IL1RA, leukemia inhibitory factor (LIF), and CCL2 (De Boeck et al., 2020). Thus, we cannot rule out the possibility that the regulation of DNA repair genes in IL33KD- or IL33oe-glioma cells could be in part due to the action of cytokines or chemokines induced by changed expression of IL-33.

While the findings related to IL-33 function in cancer progression and immunity are cumulative (Haraldsen et al., 2009; Liew et al., 2016; Cayrol and Girard, 2018), to date, evidence showing the target genes of nuclear IL-33 is limited. Despite that the regulation of DNA repair genes in glioma cells by altered expression of nuclear IL-33 is evident, the mechanisms underlying nuclear IL-33-mediated gene expression in glioma cells are largely unknown. Through examination of the glioma cell nuclear structure by TEM analysis, the appearance of cancerous nuclear characteristics, such as grooves, long clefts, and indentations (Zink et al., 2004), was lessened in IL33KD-glioma cells along with a decrease in H2A and H3 protein production. In contrast, the polylobulation and folding of the glioma cell nucleus were promoted by IL33oe in accompany by an increase in H2A and H3 proteins levels. Moreover, the nuclear IL-33 mediated the expression of non-histone chromatin-associated proteins, HMGA1 and HMGA2. The two HMGA proteins are highly expressed in many cancer types and are suggested to participate in cancer cell transformation (Ozturk et al., 2014; Sgarra et al., 2018). Since high IL-33 expression is likely correlated to progression and malignancy of glioma cells (Fang et al., 2014; Gramatzki et al., 2016; Zhang et al., 2017a; De Boeck et al., 2020), our findings suggest that nuclear IL-33 could be involved in chromatin organization and gene positioning, leading to tumor cell transformation and malignancy.

**Figure 9:**
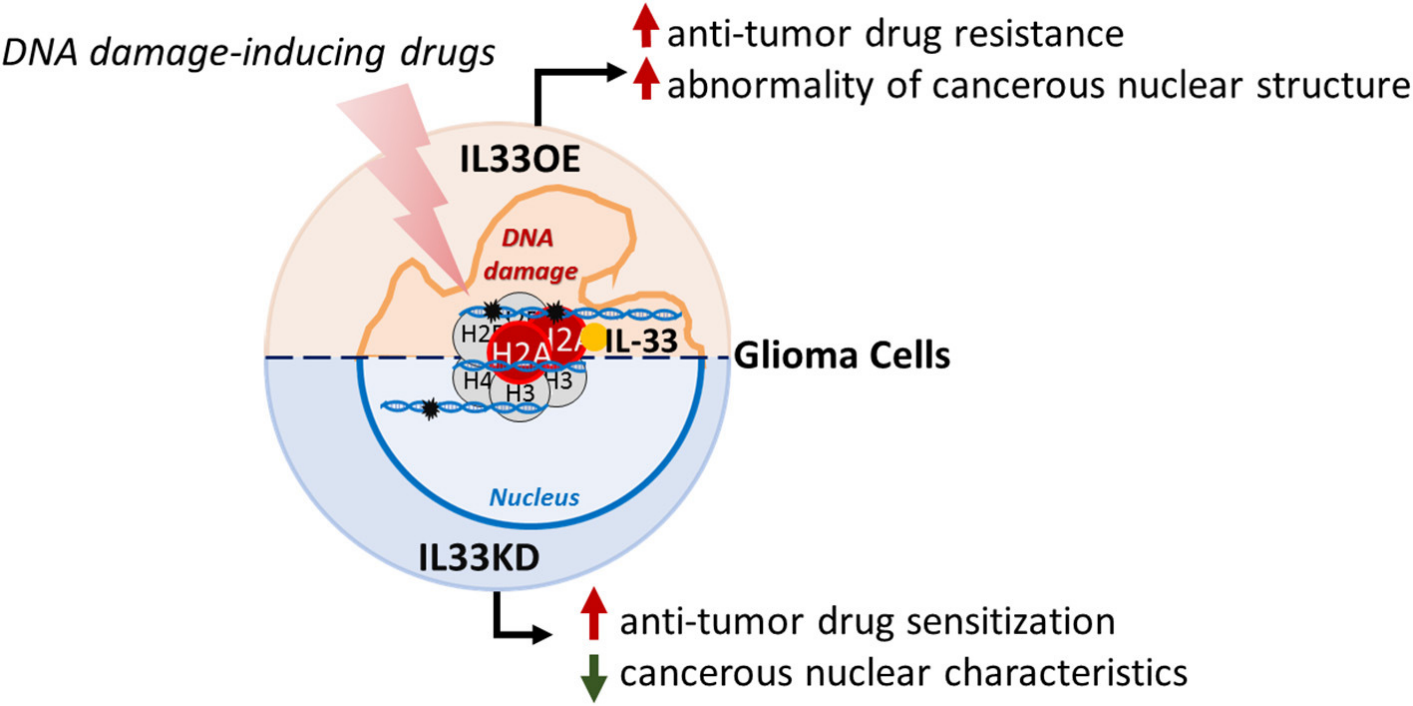
Schematic representation of nuclear IL-33 roles in glioma cells. Nuclear IL-33 interacts with H2A and is associated with chromatins in glioma cells. Declined expression of nuclear IL-33 (IL33KD) increases glioma cell sensitization to DNA damaging drugs and attenuates cancerous nuclear characteristics. In contrast, nuclear IL-33 highly expressed in glioma cells (IL33oe) increases DNA repair capability in glioma cells to induce resistance to the action of DNA damaging drugs and promotes the abnormality of cancerous nuclear structure, such as the formation of multiple nuclear lobes. IL-33, interleukin-33; IL33oe, IL-33 overexpression.

In conclusion, we provide important evidence showing that nuclear IL-33 induces resistance to the anti-tumor drug and promotes abnormal nuclear organization in glioma cells, which might contribute to glioma expansion and malignancy. The findings in this study support the view of nuclear IL-33 role in glioma progression (Figure 9).

## Data Availability Statement

The original contributions presented in the study are included in the article/Supplementary Material, further inquiries can be directed to the corresponding author/s.

## Ethics Statement

No animal studies are presented in this manuscript, and therefore no ethical approval was required.

## Author Contributions

Y-HC, QQ, and S-FT contributed to experimental designs, the interpretation of the results, and manuscript preparation. Y-HC and QQ conducted cell cultures, biochemical assays, and data analysis. W-TC assisted imaging analysis. H-YH and C-SY contributed to TEM sample preparation and TEM analysis. S-FT contributed to writing the manuscript. All authors have read the manuscript and consent the final version.

## Funding

This study was supported by grants from the Ministry of Science and Technology (MOST), Taiwan (MOST 109-2314-B-006-015-MY3).

## Conflict of Interest

The authors declare that the research was conducted in the absence of any commercial or financial relationships that could be construed as a potential conflict of interest.

## Publisher's Note

All claims expressed in this article are solely those of the authors and do not necessarily represent those of their affiliated organizations, or those of the publisher, the editors and the reviewers. Any product that may be evaluated in this article, or claim that may be made by its manufacturer, is not guaranteed or endorsed by the publisher.
