# Supplementary material for: The Nuclear Function of IL-33 in Desensitization to DNA Damaging Agent and Change of Glioma Nuclear Structure
Source: Front Cell Neurosci. 2021 Oct 20;15:713336. doi: 10.3389/fncel.2021.713336 (PMC8565524; doi:10.3389/fncel.2021.713336)
Supplement: Supplementary file 1 [file Data_Sheet_1.pdf]

Supplementary Figures

Nuclear function of IL-33 in desensitization to DNA damaging agent and change of glioma nuclear structure

Yu-Han Chung[1], Qiu Qian[1], Hsin-Ying Huang[2], Wen-Tai Chiu[3], Chung-Shi Yang[2], Shun-Fen Tzeng[1]*

[1]Department of Life Sciences, College of Bioscience and Biotechnology, National Cheng Kung University, Tainan, Taiwan

[2]Institute of Biomedical Engineering and Nanomedicine, National Health Research Institutes, Zhunan, Miaoli, Taiwan

[3]Department of Biomedical Engineering, National Cheng Kung University, Tainan, Taiwan

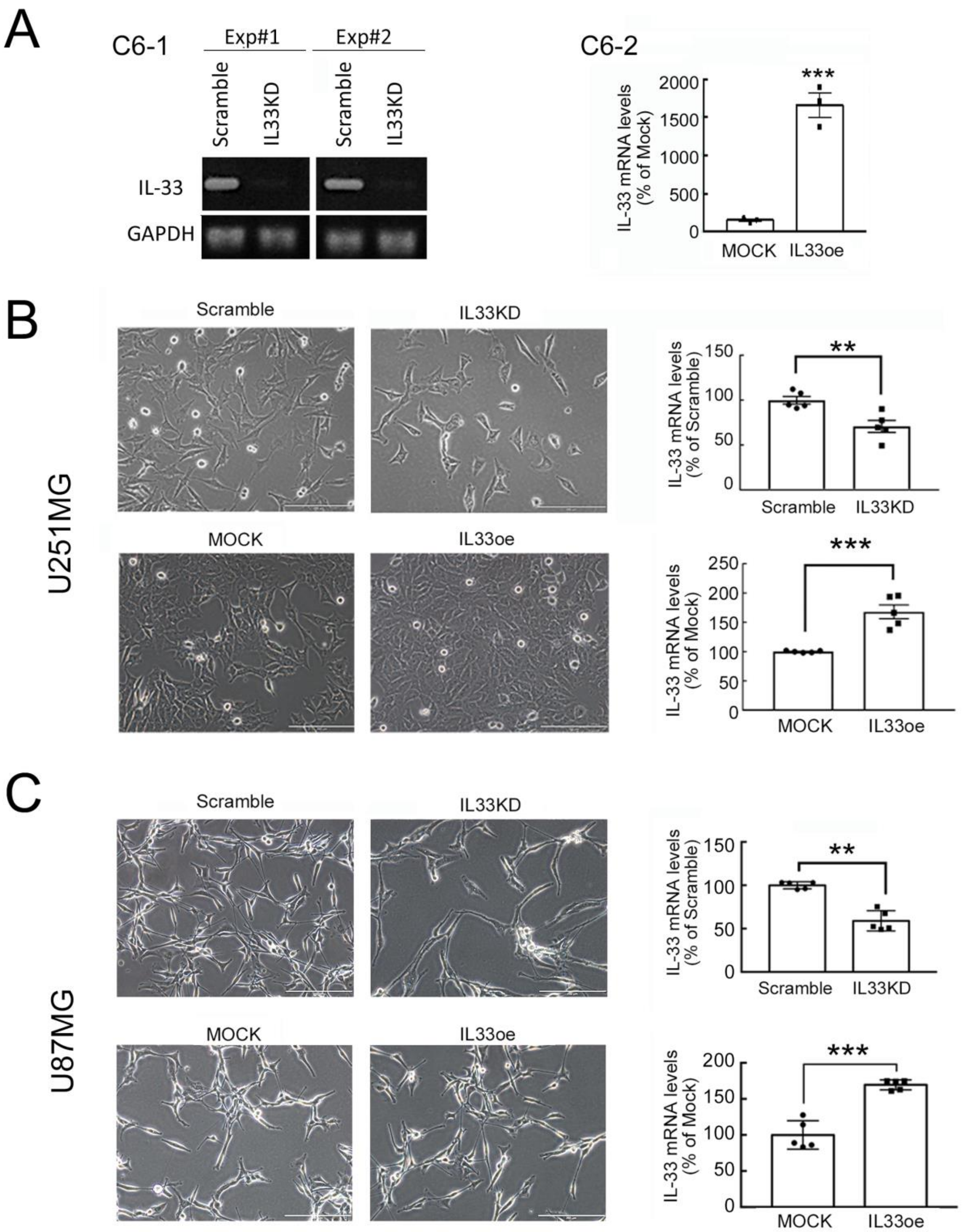

Fig. S1. Efficiency of IL-33 gene knockdown and overexpression in glioma cell lines used in this study. (A) RNA samples were isolated from C6-1 cells receiving lenti-scramble (Scramble) and lenti-shIL33_795 (IL33KD), and prepared from C6-2 cells receiving pLVpuro-EF1a-GFP-Bsd (MOCK) and pLV-EF1a-rnoIL33-IRES-Puro(IL33oe). The samples were subjected to RT-PCR analysis and confirm efficient downregulation of IL-33 in C6-1 cells (left-handed panel). The experiments were repeated from the two cell passages with similar results. Alternatively, the samples from MOCK- and IL33oe-C6 cells were subjected

to QPCR and validate IL-33 overexpression in C6-2 cells (right-handed panel). The results are present as Mean ± SEM from the three cell passages (n=3). ***P<0.001 vs. MOCK. (B, C) Human U251MG and U87MG cells were transduced by lenti-scramble (Scramble) and lenti-sh-hIL33_661 (IL33KD), as well as pLVpuro-EF1a-GFP-Bsd (MOCK) and pLVpuro-EF1a-rnoIL33-3xFLAG (IL33oe). The representative phase-contrast images after lentivirus gene transduction are shown in left-handed panel. The results from QPCR assays indicate that IL-33 expression on was significantly downregulated or increased in the two human glioma cell lines (right-handed panel). The results are present as Mean ± SEM from the five cell passages (n=5). **P<0.01, ***P<0.001 vs. Scramble (B) or MOCK (C). Scale bar, 200 μM.

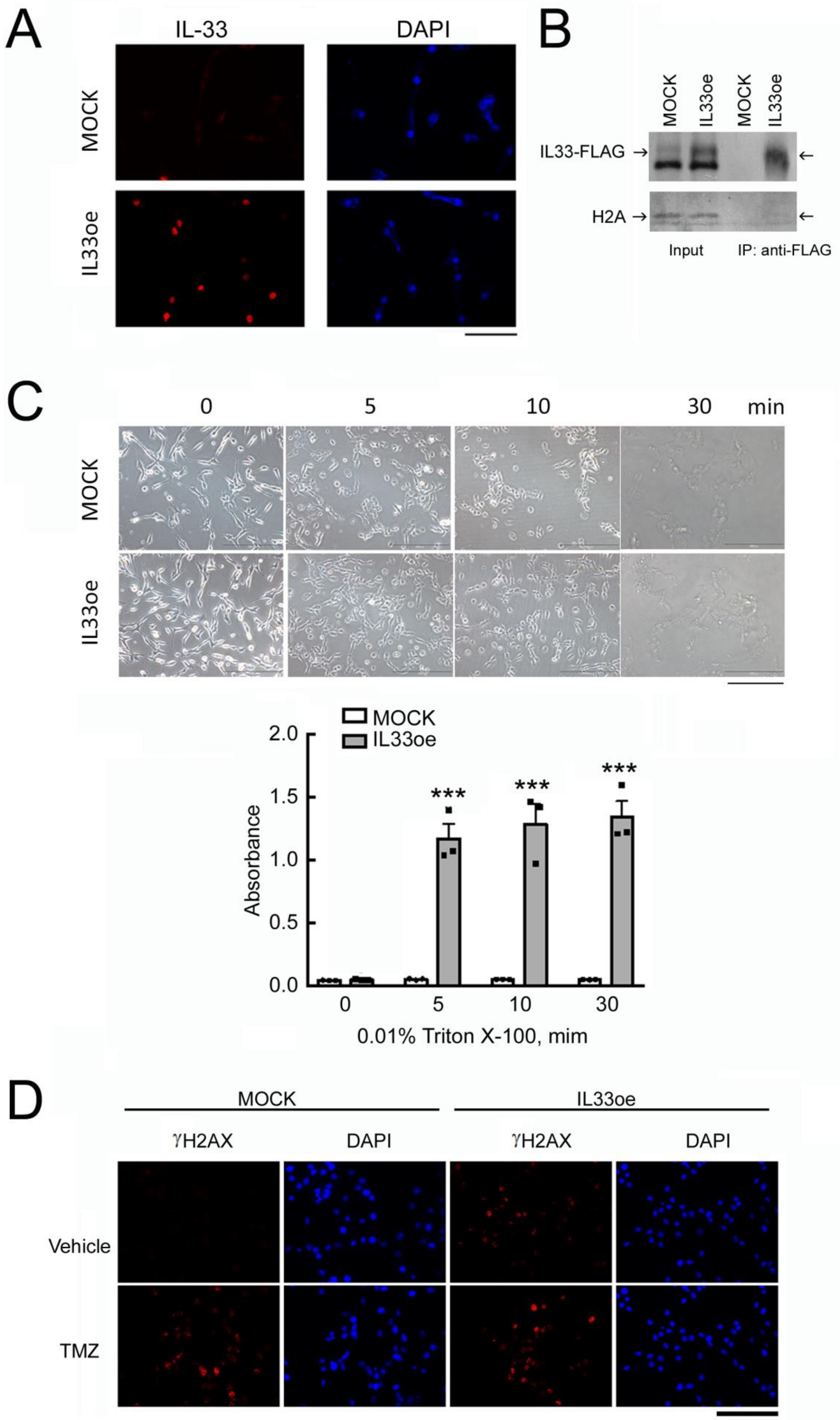

A
IL-33
DAPI
MOCK
IL33oe
B
MOCK
IL33oe
MOCK
IL33oe
IL33-FLAG →
H2A →
Input
IP: anti-FLAG
C
0
5
10
30
min
MOCK
IL33oe
MOCK
IL33oe
2.0
1.5
1.0
0.5
0.0
Absorbance
***
***
***
0
5
10
30
0.01% Triton X-100, mim
D
MOCK
IL33oe
γH2AX
DAPI
γH2AX
DAPI
Vehicle
TMZ

Fig. S2. IL-33 release in IL33oe-C6 cells by Triton X-100 and γH2AX levels in glioma cells treated by TMZ. (A) Nuclear IL-33 molecules (red) were clearly observed in IL33oe-C6 cells. IL-33 immunoreactivity was relatively weak in MOCK-C6 cells. DAPI (blue) nuclear counterstaining was conducted. Scale bar, 100 μm. (B) C6-2 cells were transduced by the control lentiviral particles (MOCK) and the lentiviral particles with IL33-FLAG (IL33oe) as described in Materials and Methods. The proteins collected by MOCK- and IL33oe-C6 cells were subjected to immunoprecipitation using anti-FLAG. Representative images were derived from the separation of total proteins (Input) or immunoprecipitates (IP) on SDS-PAGE. Arrows indicates IL33-FLAG (greater than 33kDa) and H2A (17kDa). (C) After treatment of IL33oe-C6 cells with 0.01% Triton X-100 for 5, 10, and 30 min, the culture media were collected and subjected to measure IL-33 levels using IL-33 ELISA assay kit. IL-33 was able to be detected in the culture medium starting at 5 min post treatment (bottom panel). The rounding cells with process withdrawal and cell shrinkage were observed in the cultures right after a 5 min-Triton exposure (upper panel). Cell lysis was noticed at 30 min post exposure. The results are present as Mean $\pm$ SEM from the five cell passages (n=5). ***$P<0.001$ vs. MOCK. Scale bar, 100 μm. (D) MOCK- and IL33oe-C6 cells were treated with Vehicle or 200 μM of TMZ for 72 h, and then subjected to γH2AX immunostaining (red) and DAPI nuclear counterstaining (blue). An increase in γH2AX immunoreactivity was observed in the nuclei of MOCK- and IL33oe-C6 cells treated by TMZ. Scale bar, 20 μm.

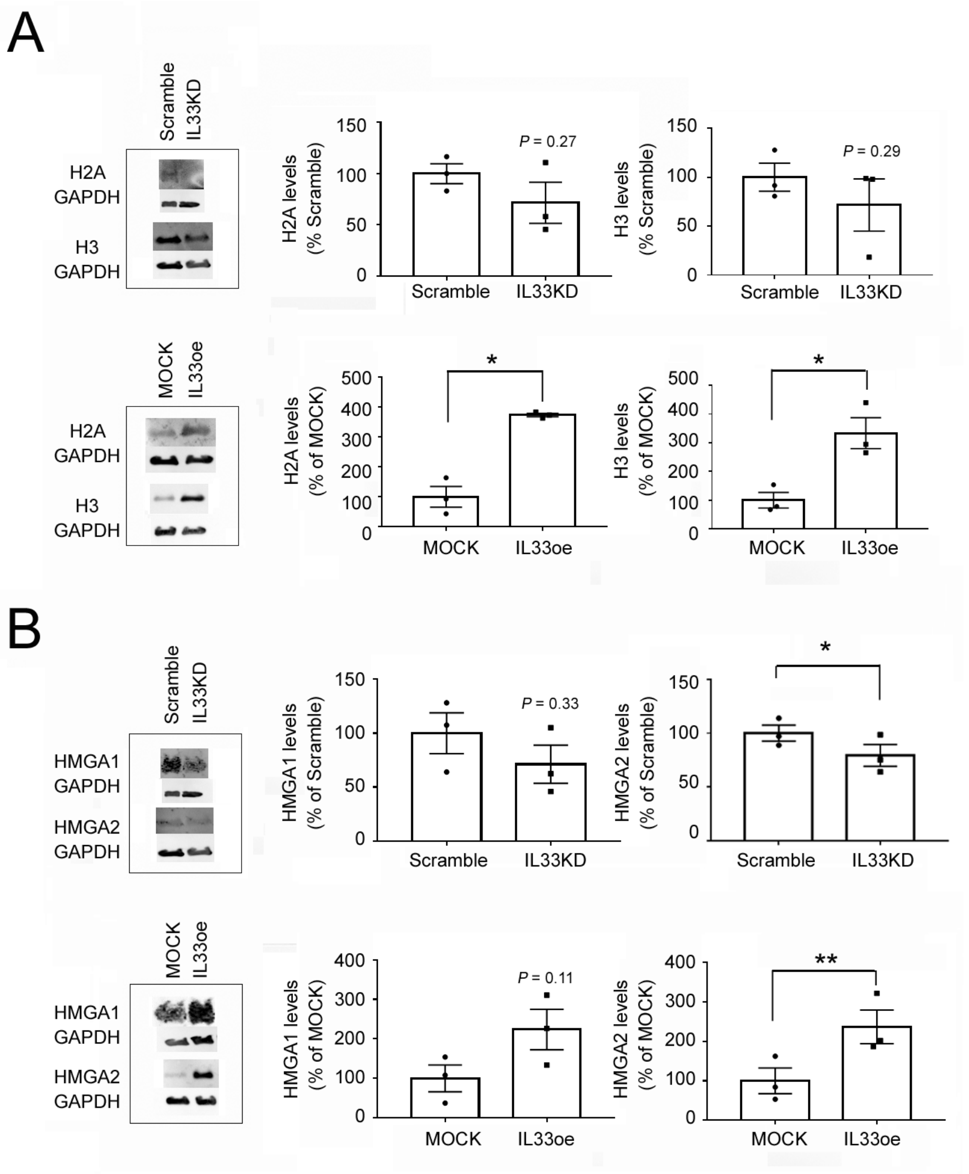

Fig. S3. **IL-33 mediates histone proteins and non-histone proteins HMGA1/HMGA2 expression in U87MG cells.** Total proteins were isolated from Scramble, IL33KD, MOCK, and IL33oe-U87MG cells and then subjected to the Western Blot analysis for the examination of histone proteins H2A and H3 (A), as well as non-histone proteins HMGA1 and HMGA2 (B). The quantification was performed by normalization of the indicated protein levels over GAPDH protein level (as a loading control). The results show that declined levels of H2A and H3 proteins were observed in IL33KD-U87MG cultures, whereas their production was increased in IL33oe-U87MG cells. In addition, the data indicate that the levels HMGA2 expression was decreased in U87MG cells by IL33KD, but increased in IL33oe-U87MG cells. The results are presented as Mean $\pm$ SEM from the three cell passages (n=3) following by the two-tailed paired Student's t-test. *P<0.05, **P<0.01 vs. Scramble or MOCK.

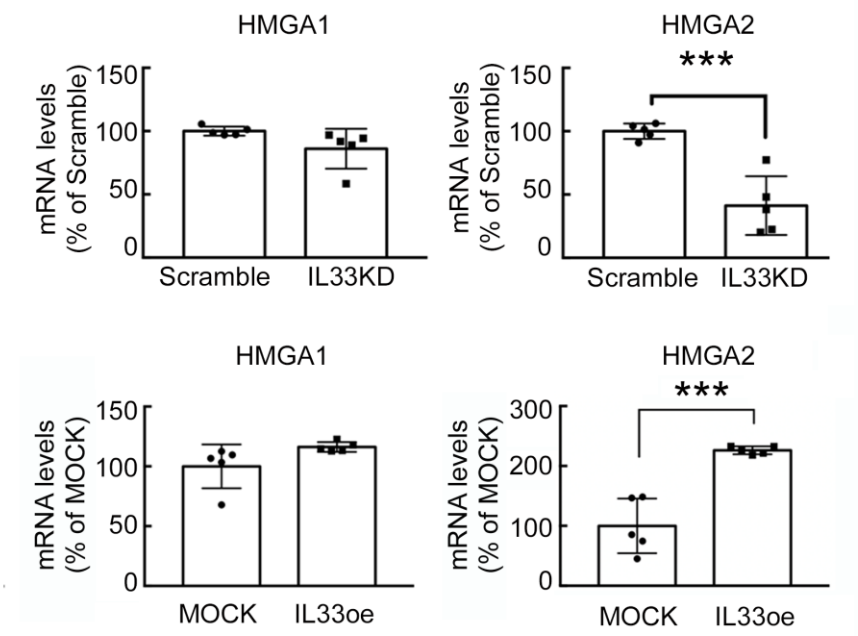

Fig. S4. IL-33 mediates non-histone proteins HMGA1/HMGA2 gene expression in U87MG cells. HMGA1 and HMGA2 mRNA expression in Scramble, IL33KD, MOCK, and IL33oe-U87MG cells was examined by QPCR analysis. The results show that HMGA2 expression was decreased in U87MG cells by IL33KD, but increased in IL33oe-U87MG cells. The results are presented as Mean ± SEM from the five cell passages (n=5). ***P<0.001 vs. Scramble or MOCK.
